# Foliar Spray or Soil Drench: Microalgae Application Impacts on Soil Microbiology, Morpho-Physiological and Biochemical Responses, Oil and Fatty Acid Profiles of Chia Plants under Alkaline Stress

**DOI:** 10.3390/biology11121844

**Published:** 2022-12-17

**Authors:** Samah M. Youssef, Rasha S. El-Serafy, Kholoud Z. Ghanem, Abeer Elhakem, Azza A. Abdel Aal

**Affiliations:** 1 Horticulture Department, Faculty of Agriculture, Fayoum University, Fayoum 63514, Egypt; 2 Horticulture Department, Faculty of Agriculture, Tanta University, Tanta 31527, Egypt; 3 Department of Biological Science, College of Science and Humanities, Shaqra University, Shaqra, Riyadh 11961, Saudi Arabia; kghanem@su.edu.sa; 4 Department of Biology, College of Sciences and Humanities, Prince Sattam Bin Abdulaziz University, Al-Kharj 11942, Saudi Arabia; a.elhakem@psau.edu.sa; 5 Soil Microbiology Department, Soils, Water and Environment Research Institute, Agricultural Research Center, Giza 12619, Egypt; prof.azzaahmed@gmail.com

**Keywords:** microalgae, *Salvia hispanica*, saturated fatty acids, linolenic acid, DPPH, cyanobacteria

## Abstract

**Simple Summary:**

Chia is an important medicinal plant and is a rich source of omega-3 and omega-6 fatty acids. Alkaline soil inhibits the growth and productivity of all crops, including chia. Microalgae are a diverse group of photosynthetic microorganisms that can be used in modest doses to stimulate the growth and productivity of numerous crops in both normal and stressed conditions. Microalgae supplementation by two application methods (foliar spray and soil drench) resulted in an increase in the growth and productivity of chia plants cultivated under alkaline stress conditions, and caused an increase in the antioxidant levels in the chia seeds, although soil drenching gained the superiority in this respect. The oil content was increased following microalgae application with an increase in omega-3 proportion. Chia plants showed different responses to foliar and drenching applications. Microalgae would be a potential and eco-friendly approach for enhancing agricultural productivity in alkaline environments. Our findings also suggest that *Arthrospira platensis* supplementation via the soil drenching technique should be used in the future to enhance plant growth and productivity under alkaline soil conditions.

**Abstract:**

Alkaline soil inhibits the growth and productivity of chia plants (*Salvia hispanica* L.). Microalgae as biofertilizers have been reported to induce alkalinity tolerance and enhance yield and quality. However, limited information is known concerning the influence of microalgae application on medical plants, including chia. Our experiments were performed to evaluate the effect of microalgae strains of *Arthrospira platensis*, *Chlorella vulgaris*, *Nostoc muscorum*, and *Anabaena azollae* with two application methods, foliar spray and soil drench, on morpho-physiological and biochemical parameters, yield, seed and oil quality, and fatty acid profiles of chia plants cultivated under alkaline soil conditions, as well as the on soil microbial activity. The results obtained reveal that both application methods positively influenced the growth and productivity of chia plants. However, the foliar application showed significant differences in the herb’s fresh and dry weights and leaf pigments, whereas the drenching application caused more effect than the foliar spray application at the reproductive stage. Untreated chia plants showed a slight decline in the growth, productivity, and antioxidant level with an increase in Na content. However, microalgae applications significantly ameliorated these impacts as they induced an enhancement in the growth, leaf pigments, total protein and carbohydrate contents, nutrient content, seed and oil yields, as well as an increase in linolenic and linoleic fatty acids, with a reduction in saturated fatty acids, namely, palmitic and lauric acid. Soil drenching generated an improvement in the soil microbial activity and caused a reduction in the pH. The treatment of *A. platensis* with drenching application resulted in higher seed and oil yield, with an increase of 124 and 263.3% in seed and oil yield, respectively.

## 1. Introduction

*Salvia hispanica* L., commonly known as chia, is a member of the Lamiaceae family and is native to the mountains of Guatemala and Mexico [1]. Currently, chia is grown in Argentina, Australia, Bolivia, Colombia, Guatemala, Mexico, Peru, and Bolivia [2]. Today, Mexico is acknowledged as the largest chia grower in the world [3]. Chia is a herbaceous annual plant that can reach a height of 1 m, has oppositely oriented leaves, and yields white or purple flowers. Chia seeds are utilized commercially as a whole or ground up to make flour, mucilage, oil seeds, and other products [4]. Furthermore, it is used as a spice in a variety of foods, including milk, yogurt, salad dressings, soups, and baked products, as well as fruit juices, due to their nutritional value. The seeds are rich in dietary fiber, minerals, proteins, vital fatty acids, carbohydrates, and polyphenolic substances [5]. Chia seed oil is a sustainable source of polyunsaturated fatty acids omega-3 (58–64%) and omega-6, the essential fatty acids for human health [6], and antioxidants, including tocopherols, phytosterols, carotenoids, and phenolic compounds such as chlorogenic acid, caffeic acid, myricetin, quercetin, and kaempferol [7]. These compounds protect consumers from a variety of diseases, and have positive effects on human health [8]. However, chia is a crop that is known to be susceptible to several forms of stress [9].

Foliar feeding is a technique of supplying plants with required nutrients by spraying an aqueous solution directly onto the leaves, which absorb these nutrients through their stomata and cuticles [10,11]. Foliar spray might be helpful when plants are lacking in a certain nutrient, but is not a substitute for healthy soil. In addition, foliar spray application avoids nutrient leaching from the soil and induces a quick response in the plant [11,12]. The soil drenching technique, on the other hand, is the process of supplying diluted products directly to the base of the plant, and has a slow impact because the chemicals applied require time to be absorbed by the roots and transported to the stems, branches, and leaves.

Soil alkalinization is a worldwide challenge that lowers agricultural quality and crop yields. Alkaline soil has a pH greater than 7.5, high CO_3_ ^−2^/HCO_3_^-^, sufficient Na levels to limit crop growth, and/or high exchangeable sodium content and/or exchange capacity (15% or more) [13]. Additionally, alkaline soil is characterized by its poor structure, surface crusting and cracking, low water holding capacity, organic matter and clay content, and loss of nutrients by leaching or deep percolation [14,15]. The main effects of alkali stress on crop production are ion toxicity and osmotic stress, but many studies report that high pH is actually more hazardous to plants than saline soil [16]. Alkaline soil can prevent seeds germinating, harm the structure of root cells, and restrict nutrient uptake, causing a reduction in agricultural yields [15,17]; biofertilization can be a useful strategy to cope with soil alkalinization. Biofertilization is a necessary strategy for environmentally friendly, sustainable farming methods [18,19,20]. Microalgae have attracted great attention due to their potentially extensive application in agriculture as biofertilizers. Microalgal biofertilizers may be employed in crop production to improve agricultural sustainability [21].

Microalgae are a diverse group of photosynthetic microorganisms that can be used in modest doses to stimulate the growth and development of numerous crops in both normal and stressed conditions. They can be used in concert with synthetic fertilizers to control plant growth, protect crops, increase yields, and support plant tolerance to environmental stresses [22]. Microalgae are renewable, sustainable, and economical sources of bioactive pharmaceutical products, biofuels, and food ingredients [23,24]. Microalgae are microscopic single cells that can be either eukaryotic, such as green algae (Chlorophyta), or prokaryotic, such as cyanobacteria (chloroxybacteria). Cyanobacteria have been successfully applied as biofertilizers in soil restoration techniques for alkaline and calcareous soils [25]. Microalgae have the capacity to promote plant growth, immobilize heavy metals in the soil, link soil particles into stable aggregates, and lessen the likelihood of erosion [25]. Microalgae biofertilization for rice plants is related to their ability to fix nitrogen, and has other beneficial effects for both plants and soil [24]. *Chlorella* sp. cell soil application with irrigation water enhanced the chlorophyll level and dry weights in cucumber, rice, eggplant, and lettuce crops [26].

To our best knowledge, the effects on *Salvia hispanica* L. treated with foliar spray and soil drenching application methods using different microalgae strains under alkaline soil conditions are reported here for the first time. The aim was to evaluate the differential impact of different microalgae strains, used in the field under alkaline conditions, on chia plants under different application modes. To achieve this goal, we evaluated the influence of *C. vulgaris*, *N. muscorum*, *A. platensis*, and *A. azollae* strains on the growth and productivity of chia, including photosynthetic pigments, antioxidant activity, yield, and yield components. Additionally, we assessed the effects of microalgae strains on the seed quality (oil content and fatty acid composition) and leaves, as well as soil-related traits.

## 2. Materials and Methods

### 2.1. Location and Soil Analysis

In a private nursery in El Fayoum Governorate, Egypt, between latitude 29^°^19′30.1″ N and longitude 30^°^51′15.8″ E, two experiments were performed during the cropping seasons of 2019/2020 and 2020/2021, in the period of November to July for both seasons. Prior to the experiment, the soil physiochemical parameters were evaluated in accordance with the methods described by Page et al. [27] and Klut [28]. The soil biological parameters were assessed using the technique of Allen [29] and Allen and Stanier [30], and the results are presented in Table 1. The soil class was identified as alkaline based on the soil characteristics according to Oster and Ayawardane [31].

### 2.2. Plant Material and Cultivation 

Chia seeds were acquired from the Faculty of Pharmacy’s Experimental Farm at Cairo University. The seeds were sown in trays on 5 October for both seasons. The symmetrical seedlings with 2–3 pairs of truly extended leaves and a length of 10 to 15 cm were planted out in the open field in plots on 20 November for both seasons. The experimental plot had a total area of 4.8 m^2^ (8 m in length and 0.6 m in width), and roughly 0.3 m in inter-seedling distance within each row.

### 2.3. Microorganisms Source and Culture 

The green alga *C. vulgaris* and three cyanobacteria, namely, *N. muscorum* and *A. platensis*, as well as an *A. azollae* strain, which was isolated from *Azolla pinnata* [23], were obtained from the Microbiology Department, Soils, Water and Environment Research Institute, Egypt. Both *N. muscorum* and *A. azollae* were grown on nitrate-free medium (Blue Green “BG”11 medium) according to Rippka et al. [32], while *A. platensis* was cultivated on Zarrouk medium according to Zarrouk [33]. *Chlorella vulgaris* was cultured in a Bold medium [34]. The cultures were incubated in a growth chamber under continuous illumination (24 h light, 2000 lux) and a temperature of 25 °C ± 2 °C for all strains except the mesophilic alga *A. platensis*, which was grown at 35 °C ± 2 °C. After 30 days of incubation, the algal suspension was used for the field experiments. To determine algal biomass, 100 mL of the algal culture was taken after 30 days of incubation, centrifuged at 10,000 rpm for 10 min, and the supernatant was discarded. The pellet was centrifuged for 10 min at 10,000 rpm after passing through three phases of resuspension in distilled water to wash it. The cleaned pellet was weighed, air dried; then, the dry weight was recorded and calculated as g/L. For the field experiments, 1 g/L of dried algal biomass was used.

### 2.4. Treatments and Experimental Design

The experimental configuration had a split-plot system built on a randomized complete block design (RCBD) with three replicates. The application method and microalgae inoculations were distributed at random across the main and subplots, respectively. The experiments consist of ten different treatments, applied as interactions of two application methods (foliar spray and soil drench) and microalgae treatments (untreated control, *A. platensis, C. vulgaris*, *N. muscorum,* and *A. azollae*). The algal growth suspension was used for foliar and soil drenching application at a rate of 50 L ha**^−^**^1^, divided into two equal doses [35]. The treatments were performed twice at 40 and 60 days after seedlings were planted. Other agricultural practices (fertilization, irrigation, and weeding) were carried out exactly as per Souza and Chaves [36].

### 2.5. Morpho-Physiological Traits 

Seven plants aged 90 days were collected at random from each replicate to determine the fresh and dry weights of the herb (g/plant). Total chlorophyll was assessed from the top third and fourth leaves using a chlorophyll meter (SPAD-502, Minolta Company, Tokyo, Japan) [37]. The dimethylformamide (DMF) technique was used to estimate total carotene content (mg/mm^2^) [38]. 

### 2.6. Yield and Yield Components

At the harvesting stage, seven plants were collected to determine the number of inflorescence plant**^−^**^1^, seed yield plant**^−^**^1^ (g), weight of 1000 seeds (g), and seed yield ha**^−^**^1^ (t). The oil content in the chia seeds was determined following the same steps described in the method of AOAC [38]. Then, oil percent and oil yield ha**^−^**^1^ (L) were calculated. Oil (%) was expressed using the following formula:

Oil (%) = (Weight of extracted oil (g) ÷ sample of seed weight (g)) × 100.(1)

### 2.7. Gas–Liquid Chromatography (GLC)

Gas–Liquid chromatography (GLC) (Hewlett-Packard Corporation, GA, USA) was utilized to identify the fatty acid composition of the oil samples [39]. Methylated fatty acids were fed into the HP6890 Series GC system using a column of type DB-23 (60 m × 0.32 mm × 0.25 µm). Nitrogen was used as the carrier gas, flowing at a rate of 1.5 mL/min and having a splitting ratio of 1:50. The temperatures of the injector and flame ionization detector (FID) were 250 °C and 280 °C, respectively. The following were the temperature settings: 150 °C to 210 °C at a rate of 5 °C per minute, followed by a 25 min hold at 210 °C. Peaks were located by contrasting the obtained retention durations with standard methyl esters.

### 2.8. Biochemical Analysis 

Total protein and carbohydrate contents, as well as total phenols, total flavonoids, and DPPH levels in chia seeds were estimated. Total protein was assessed using the semi-micro Kjeldahl technique [39]. Total carbohydrates were measured colorimetrically as previously described by Alshallash et al. [40] using the phenol–sulfuric acid technique [41]. For extracting total phenolics and DPPH in the seeds, 1 g of seed samples was mixed with 10 mL of ethanol (60%) and left for 3 h under shaking conditions at room temperature; then, the mixture was centrifuged at 3000× *g* for 15 min. Total phenolics were estimated by the Folin–Ciocalteu colorimetric technique [42]. Briefly, 0.1 mL of the extract was diluted with 2 mL of distilled water, and then 0.5 mL of the Folin–Ciocalteu reagent was mixed. A 2 mL solution of sodium carbonate (20%) was added to each tube. Evaluating the test solutions’ absorbances at 650 nm was conducted in comparison to the reagent used as a blank. Total flavonoids determination was assessed using the Shi et al. [43] method with minor modification according to Zhao et al. [44]. Briefly, 1.5 mL of the ethanol extract was mixed with 4.5 mL of distilled water and 1 mL of 5% sodium nitrite solution in a 25 mL tube. Subsequently, 1 mL of 10% aluminium nitrate solution was added to the mixture after 6 min. Following the addition of 10 mL of 4% sodium hydroxide solution after 6 min, 25 mL of 60% aqueous ethanol was added. The mixture**’**s absorbance at 510 nm was measured after it had been reacting for 15 min in comparison to a blank that contained 5 mL of extraction solvent. The capacity of seed extract to scavenge free radicals was evaluated using the DPPH (2,2-diphenyl-1-picrylhydrazyl) technique [45]. Next, 33 μL of the ethanol extract and 1.3 mL of the diluted-in-methanol (0.024 mg/mL) DPPH solution were combined. The absorbance at 515 nm was measured following 30 min of dark incubation. 

### 2.9. Nutrients Determinations

Macroelements, i.e., phosphorus (P), potassium (K^+^), and calcium (Ca^2+^), as well as micronutrients, i.e., iron (Fe^2+^), magnesium (Mg^2+^), zinc (Zn^2+^), and sodium (Na^+^), were measured in the seeds. The chlorostannous molybdophosphoric blue color technique applied in the sulphuric acid method was used to determine P content [46]. The K and Na levels were measured using a flame photometer (Gallenkamp Company, London, UK) [47]. The Ca and micronutrient (Fe, Mg, and Zn) contents were assessed using an atomic absorption spectrophotometer (Perkin-Elmer, Model 3300, Woodbridge, Canada) [48].

### 2.10. Soil pH and Microbial Count 

During harvesting, when applying the microalgae as a drenching application, the pH of the soil was estimated using the techniques of Page et al. [27]. The bacterial and total cyanobacteria count were determined according to Allen [29] and Allen and Stanier [30], as follows: mixed soil samples from different sites in each treatment were collected, using a knife, from a depth of 0–10 cm and placed into a plastic container under sterile conditions. The dilution plate method was used to calculate the total count of bacteria. The total bacterial count was determined on Thornton’s medium, amended with 50 mg of pentachloronitrobenzene, 40 mg of actidione, and 35 mg of pimaricin, then incubated at 30 °C. The total cyanobacterial count was calculated by mounting tenfold serial soil suspension-dilutions in triplicate onto an agar-coated BG11 medium. The MPN tubes were incubated at 25 °C in the presence of light (50 µmol photon m**^−^**^2^ s**^−^**^1^). 

### 2.11. Statistical Analysis

Prior to performing two-way analysis of variance (ANOVA), the homogeneity and Shapiro–Wilks normality test of error variance for all variables were investigated. The results showed that all the data were homogeneous enough to support additional ANOVA testing. The combined analysis was carried out for all variables utilizing ANOVA based on the split-plot system in RCBD using the InfoStat computer program (version 2020b). Using Duncan’s multiple range as a post hoc test (DMRT) at *p* ≤ 0.05, the variable means were compared [49]. Mean values are presented as mean ± standard error.

## 3. Results

### 3.1. Morpho-Physiological Traits

The herb weights and leaf pigment values presented in Table 2 reveal that chia plants exposed to foliar application with different microalgae strains showed significant differences in the herb fresh and dry weights, and in leaf pigments, relative to the soil drenching technique. Herb fresh and dry weights as well as total chlorophyll and carotenoids of foliar-sprayed plants exhibited 4.26, 4.76, 3.81, and 16.28% increases as compared with plants treated with soil drenching, respectively. Microalgae strains also considerably affected the vegetative parameters, viz., herb fresh and dry weights and total chlorophyll and carotenoids, and treatment with *A. platensis* significantly resulted in the highest values, followed by *C. vulgaris* and *N. muscorum*. By the mean of the interaction treatments, the foliar spray with *A. platensis* was the most effective treatment, when compared to other treatments, as the plants significantly revealed the highest values in this respect, while the lowest values were produced by untreated control plants. 

### 3.2. Yield and Yield Components

**Figure 1:**
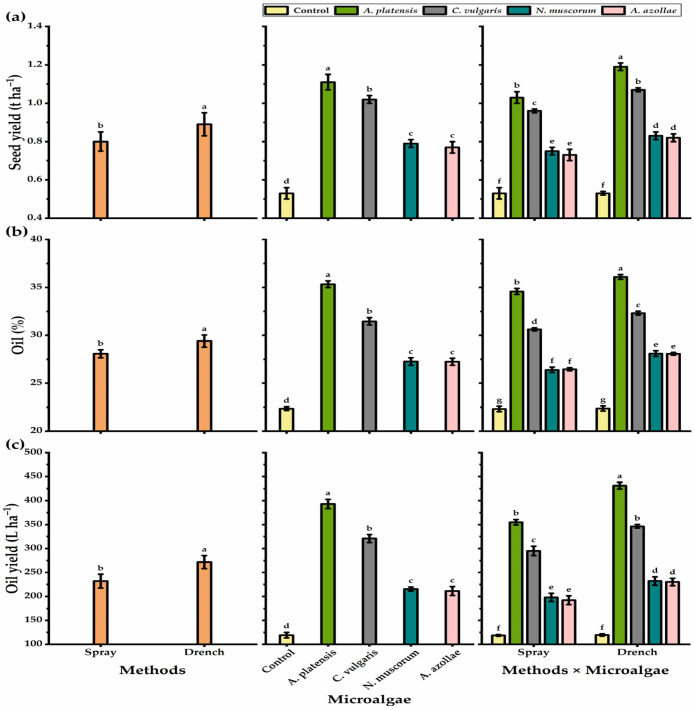
Seed yield ha ^−1^ (t) (**a**), oil %^−1^ (**b**), and oil yield ha^−1^ (L) (**c**) of chia plants cultivated under alkaline stress conditions in response to different application methods, microalgae strains, and their interaction. The bars with different letters are significantly different at *p* ≤ 0.05 by DMRT. Mean values are presented as mean ± standard error.

The results presented in Table 3 show the yield and yield component responses of chia plants cultivated under alkaline conditions to microalgae treatments with two application methods. The yield and yield component traits were impacted by season, method, microalgae, and their interactions (Table 3, Figure 1). Number of inflorescence plant^−1^ showed a different trend when compared with other yield component traits (1000 seed weight, seed and oil yields), as chia plants that received foliar spray with microalgae showed a significant increase in their inflorescence number plant^−1^ relative to plants treated with soil drenching (Table 3). Foliar-sprayed plants with *A. platensis* showed the highest number of inflorescence plant^−1^, although the differences with the rest treatments were not significant.

Regarding the traits of 1000 seed weight, seed yield plant^−1^, and seed yield ha^−1^, the plants exposed to the drenching application presented higher values than the plants treated with the foliar spray method, as the former method resulted in 2.69% higher 1000 seed weight and 10% increases in both seed yield plant^−1^ and seed yield ha^−1^. Chia plants subjected to *A. platensis* showed a significant increase in the seed yield plant^−1^ (52%), 1000 seed weight (51.4%), and seed yield ha^−1^ (52.3%) as compared with those of non-microalgae-treated plants. Concerning the *N. muscorum* or *A. azollae* strains, they did not differ significantly for all yield parameters except 1000 seed weight. All interaction treatments showed a significant increase in all seed traits as compared with untreated controls. However, the soil drenching method with *A. platensis* was the most efficient of all interactions.

Concerning oil percent and oil yield, drenching treatment significantly increased oil percent and oil yield traits, as it induced a 4.49 and 14.72% increase as compared with foliar treatments, respectively (Figure 1). The highest oil yield of chia seeds was obtained by the *A. platensis* microalgae strain; among the microalgae strains, *N. muscorum*- and *A. azollae*-treated plants showed the lowest oil percent and oil yield, with a non-significant difference between them. On the other hand, untreated plants significantly recorded the lowest values in this respect (22.4% and 119 L for oil percent and yield, respectively). The soil drenching treatment with *A. platensis* outperformed interactions, and the interaction treatments significantly increased oil characteristics as compared to untreated controls.

### 3.3. Fatty Acid Profile

The results presented in Table 4 reveal that all treatments modified the bioactive ingredient proportion of chia oil. Under alkaline stress conditions, the interaction between different methods and microalgae showed twenty compounds in the extracted oil, and all interactions resulted in an increase in all components of the oil. Total saturated fatty acids (ranging from 6.51 to 11.81%) contained mainly lauric, myristic, pentadecanoic, palmitic, margaric, stearic, docosanoic, and tetracosanoic acids, with a predominant proportion of palmitic acid (C16:0). The highest percentage of total saturated fatty acids (7.68%) was found in the oil of chia plants supplied with *C. vulgaris* soil drench. Palmitoleic, oleic, arachidic, and gadoleic fatty acids accounted for most of the total monounsaturated fatty acids (ranging from 7.01 to 20.52%). *Anabaena azollae* soil-drenching-treated plants contained the highest percentage (15.59%) of oleic acid (C18:1 n-9). Total polyunsaturated fatty acids ranged from 72.42 to 86.23%, with the majority of linoleic (C18:2 n-6), α-linolenic (C18:3 n-3), and γ-linolenic (C18:3 n-6) acids, whereas plants treated with *Arthrospira platensis* soil drenching had the highest percentage of this fatty acid (86.2%). Linoleic (C18:2 n-6) and γ-linolenic (C18:3 n-6) acid constituted the group of n-6, whilst n-3 was represented by α-linolenic acid (C18:3 n-3). The ratio of n-6 to n-3 was determined to be in the range of 0.30 to 0.40. Plants treated with *Arthrospira platensis* foliar spray had the greatest n-6: n-3 ratio (0.40).

### 3.4. Biochemical Traits 

The total protein, total carbohydrate, total phenolic, total flavonoid, and DPPH contents of chia plants in response to microalgae supplementation in two different application methods under alkaline stress conditions are presented in Table 5. The drenching method considerably outperformed the spray method for total protein, total carbohydrate, total phenolic, total flavonoid, and DPPH contents by 5.39, 3.88, 5.80, 12.1, and 2.61%, respectively. Furthermore, microalgae strains significantly outperformed the untreated controls in terms of total carbohydrate, total phenolic, total flavonoid, and DPPH contents (21.2, 109.9, 139.6, and 16.8% for *A. platensis*, and 11.2, 91.5, 101.2, and 12.5% for *C. vulgaris*, respectively). In contrast, application of *N. muscorum* and *A. azollae* achieved the highest total protein content in chia seeds in comparison to the control and the other microalgae strains. The chemical composition and antioxidant content of chia seeds were both considerably raised by all interactions between the application techniques and microalgae. In comparison to the rest of the interactions, the *A. platensis* soil drenching treatment substantially (*p* ≤ 0.01) induced an increase in total carbohydrates (24.9%), total phenolics (114.7%), total flavonoids (152.6%), and DPPH (18.1%), while application of the *N. muscorum* or *A. azollae* soil drenching resulted in an increase in the total protein content in seeds by 56.8 and 57.4%, respectively. Foliar-sprayed plants with *A. platensis* were placed in the second rank in this respect, as they exhibited 40.1%, 43.7 mg 100 g^−1^, 22.3 mg 100 g^−1^, and 70.2 μg mL^−1^ for total carbohydrates, total phenolics, total flavonoids, and DPPH. Among all microalgae applications, foliar application with *N. muscorum* recorded the lowest values in this respect. 

### 3.5. Nutrient Content

The results in Table 6 show that microalgae supplementation with two methods of application had a significant impact on the uptake and accumulation of K^+^, Ca^2+^, Fe^2+^, Mg^2+^, Zn^2+^, and Na^+^ in comparison to untreated plants. Mineral content of K^+^, Ca^2+^, Fe^2+^, Mg^2+^, and Zn^2+^ in chia seeds collected from soil-drenching-treated plants were substantially higher than those obtained from foliar-sprayed plants by 6.75, 11.4, 7.79, 12.3, and 6.33%, respectively, while Na^+^ content in the seeds was lower by 5.47%. In contrast, P content in seeds did not differ significantly between both methods. Statistical significance variations between the microalgae treatments were noted for P, K^+^, Ca^2+^, Fe^2+^, Mg^2+^, Zn^2+^, and Na^+^. The highest macro- and micronutrient levels were obtained by *A. platensis*-treated plants. In contrast, Na^+^ content in seeds was dramatically reduced by the microalgae application as compared to the control plants. Drenching plus *A. platensis* was substantially more efficient than spray with control, resulting in significant increases in the levels of K^+^, Fe^2+^, Mg^2+^, and Zn^2+^ by 82, 78.2, 104.1, and 63.1%, respectively. Meanwhile, foliar-sprayed plants with *N. muscorum* exhibited the lowest nutrient content as compared with other microalgae applications. The lowest Na value was produced by chia plants foliar sprayed with *A. platensis*, being 57.8% lower as compared with control plants.

### 3.6. Soil pH and Microbial Count 

**Figure 2:**
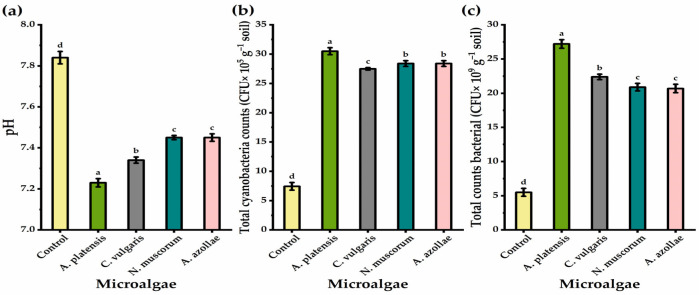
pH (**a**), total cyanobacteria (**b**), and total bacterial count (**c**) of soil cultivated by chia plants under alkaline stress conditions in response to soil drenching application method with microalgae treatments. The bars with different letters are significantly different at *p* ≤ 0.05 by DMRT.

Results illustrated in Figure 2 show that soil treated with microalgae via the drenching method resulted in significant differences in the total counts of soil microorganisms (bacteria and cyanobacteria) and the pH values, as compared to those of non-microalgae-treated plants. The soil pH value was significantly decreased relative to the control; effects were greatest with the application of *A. platensis*, followed by *C. vulgaris*, *N. muscorum*, and *A. azollae*, via drenching (by 8.44, 6.81, and 5.23%, respectively). Likewise, the most noticeably increasing bacteria counts was shown in the application of *A. platensis*, followed by *C. vulgaris* and *N. muscorum,* via drenching. The greatest significant increases in cyanobacteria counts were observed with the application of *A. platensis*, followed by *N. muscorum* or *A. azollae*, and then *C. vulgaris*, as a soil drench.

## 4. Discussion

### 4.1. Growth and Yield Attributes 

Climate change and inadequate farming practices have caused agricultural land to become more alkaline, threatening the sustainability of environmental quality and food production. One of the suggested remediation strategies is to use effective microalgae with different application methods. These factors might make microalgae an essential part of an agricultural production strategy for controlling alkalinity. Our results reveal that all microalgae strains significantly improved chia growth and yield. Microalgae can stimulate plant growth through atmospheric nitrogen fixation [50], increasing IAA, gibberellin, and cytokinin levels in plants [51,52,53]. Microalgae are used as biofertilizers because of their high content of bioactive components, including pigments (chlorophyll a, b, β-carotene, phycobilin, phycoerythrin, and xanthophyll), phenolics, peptides, and lipids [54], as well as high protein content and levels of micronutrients, polyamines, natural enzymes, carbohydrates, amino acids, and vitamins [55], all of which affect overall plant metabolism, synthesis of photosynthetic pigments, and enzymatic activity, causing an improvement in plant growth and productivity. Furthermore, microalgae contributed to an increase in endogenous hormone content, which is responsible for branch development, postponing leaf senescence, and floral transition [56]. Under stress conditions, microalgal can alter certain biochemical processes to produce antagonistic compounds, leading to plant tolerance [57]. The favorable seed and oil yields obtained in the current study may be due to the positive effect of microalgae as a high-value organic and growth regulator [58]. Additionally, this may be because microalgae have a positive effect on relative water content and nutrient status [59], increasing the ability for leaf metabolism, cell elongation, and expansion [60], as well as providing effective regulation of primarily the zinc finger protein-160 [61]. 

**Figure 3:**
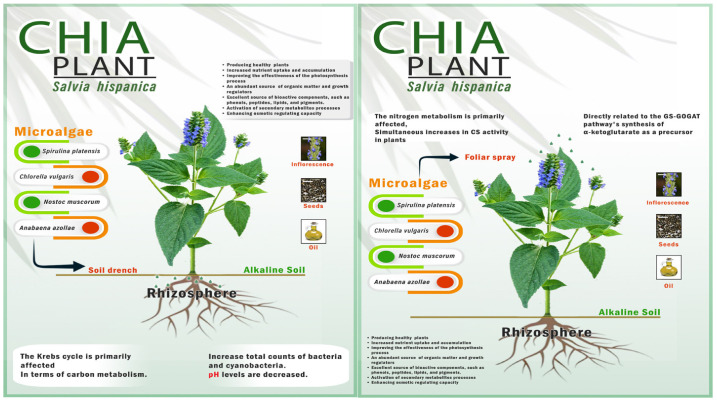
The mechanisms of foliar spray and soil drenching with microalgae supplementation on chia plants cultivated under alkaline stress conditions.

The present report reveals better performances in herb fresh and dry weights, inflorescence number, and leaf pigments by foliar application with *A. platensis* than with other strains. The drenching treatments with the same microalgae strain showed a biostimulant effect on chia plants, having a significant impact on all yield component traits. The biostimulating effect of foliar application on the growth has already been shown clearly at the vegetative stage (Table 2), whereas at the reproductive stage, the drenching application was more effective than the foliar spray application. These results indicate that each application method has quite different mechanisms of action (Figure 3). Regarding the foliar spray treatment, foliar sprays quickly provide plants with maximal nutrient absorption and utilization [62]. Additionally, based on literature data, it appears to primarily affect nitrogen metabolism, with simultaneous increases in citrate synthase activity in plants, which may be directly related to the GS-GOGAT pathway’s synthesis of α-ketoglutarate as a precursor [63]. Soil drenching application provides plants with the required nutrients slowly, enhances the nutrient availability in the root zone, and improves the yield traits [64]. Soil drenching appears to primarily affect the Krebs cycle in terms of carbon metabolism, as seen by the simultaneous increase in citrate synthase and malate dehydrogenase enzymes [56]. These results are in line with the findings obtained by Mohamadineia et al. [65], Faheed and Abd-El Fattah [66], Agathokleous et al. [67], Puglisi et al. [68], and Suchithra et al. [69], but disagree with Li et al. [70], who found that foliar application of microalgae extract was more successful than soil drenching in promoting the growth and quality of seeds. 

### 4.2. Oil and Fatty Acid Profiles

The current study also demonstrates that the application of different microalgae with both methods caused a considerable increase in the oil percentage and fatty acid composition in chia seeds. These traits were positively correlated with increased growth, nutrition uptake, and biochemical determination. Chia oil’s fatty acid composition is made up of a combination of saturated and unsaturated fatty acids, which are further divided into monounsaturated and polyunsaturated fatty acids depending on the number of unsaturated bonds. The increase in oil content and fatty acid composition may be credited to microalgae**’**s capacity to synthesizes lipids, including a wide range of fatty acids [71], particularly long-chain polyunsaturated fatty acids from the omega-3 and omega-6 groups and carotenoids such as β-carotene, astaxanthin, and lutein [72]. Additionally, linoleic, linolenic, and palmitic acids, which are frequently found in chia oil, made up the majority of the fatty acids found in the isolated microalgae lipid [73,74].

In addition, the application of microalgae with both methods under alkaline conditions increased the ratio of omega-6 to omega-3. The difference in the positions of the first double bond in the carbon chains of fatty acids (n-3 and n-6 PUFA) is the cause of the stark disparities in their biological roles that may result from the course of their interactions [75]. The medicinal effectiveness of microalgae is commonly acknowledged to depend on these components [76]. Its anti-inflammatory and antioxidant effects are significant [77]. The microalga and its separated chemicals can have the following effects: anti-neural [78], anti-liver toxicity [79], anti-diabetes, immunomodulatory [80], cardiovascular disease prevention, and anticancer [81]. Hence, the significance of the seeds as a result of these effects in terms of medicine, food, and food supplements is high, helping to improve the general health of humans.

### 4.3. Physiological and Biochemical Analysis

Leaf pigment levels in chia plants showed values significantly higher than the respective controls at all sampling treatments. Leaf chlorophyll content is an indicator of overall plant growth as it is critical for photosynthesis, which provides energy for plant growth, metabolism, and reproductive activities [82]. Carotenoids are another form of light harvesting system that also showed an increase in content (Table 2), stimulating the photosystems’ capability to perform light interception and transfer the energy absorbed to the reaction centers [82]. Carotenoids are significant antioxidant pigments that play a crucial role in photosynthesis as well as plant disease defense [51].

Carotenoids make up most of the pigments in the antenna complexes. In plant leaves, the level of chlorophyll is an indicator of how N is distributed [83]. A significant improvement in protein content occurred in chia plants treated with *N. muscorum* and *A. azollae* rather than other microalgae strains. *Nostoc muscorum* has the capacity to fix atmospheric nitrogen and a good source of protein [50]. Additionally, *Anabaena* is a rich source of protein and necessary amino acids [84]. Similarly, foliar and soil applications positively influenced the protein content. This improvement in protein content is probably essential to support the enhanced growth of microalgae-treated plants [82]. The increase in fresh and dry leaf weights in treated chia plants was likely related to the increased content of soluble compounds (pigments and proteins) in the leaves, as evidenced by the strict relationship between carbon fixation and solar radiation absorption [85]. Likewise, significant increases in antioxidants (phenols, flavonoids, and DPPH) were seen in response to the application of microalgae with different methods under alkaline stress, as compared to non-microalgae treated (Table 4). This may be attributable to the beneficial effects of microalgae as a biofertilizer [86], which also led to a rise in the activity of enzymes that help plants absorb nutrients, including nitrate reductase, dehydrogenase, alkaline or acidic phosphatase, and activity of ROS-scavenging enzymes such as catalase [87]. These parameters’ substantial increases could be explained by the fact that they are secondary metabolites [88]. Proteins, carbohydrates, and antioxidants as osmolytes and osmoprotectant compounds are essential for controlling osmotic pressure during alkaline stress conditions [89,90]. In addition, using various microalgae application methods to cultivate chia plants under high-alkalinity conditions, the accumulation of these osmolytes improved the plant’s ability to withstand alkalinity stress through the biosynthetic regulation of osmolytes and certain plant hormones [91]. Osmolytes’ beneficial impact on stressed chia plants may be due to their function in maintaining membrane stability and reducing plant cell physiological dehydration [92]. A similar pattern of osmolyte accumulation under varied stresses was reported by Tran et al. [93], Gr et al. [94], Singh et al. [95].

### 4.4. Nutrient Content 

Microalgae improve the soil’s quality, resulting in healthy plants and increased nutrient uptake and accumulation, thus increasing productivity [25]. Alkaline-stressed plants treated with various microalgae applications via drenching or spraying showed lower Na^+^ concentrations in seeds than untreated plants, which may indicate that the microalgae precluded Na^+^ uptake by roots and prevented their transfer to shoot tissues [96]. Even though non-selective K^+^ channels make it simple for Na^+^ to enter plant cells, K^+^ is most often used in the cytoplasm to modify the osmotic pressure under alkalinity-related situations [97]. Contrarily, the significant increases in macro and micronutrient contents observed after a variety of microalgae application methods under alkalinity stress conditions may be attributable to the membrane’s resilience by promoting the H+ -ATPase activity in the cell membrane [98], which increases the uptake of K and Ca nutrients and enhances the uptake of water and photosynthesis [52]. Similarly, Ertani et al. [99] observed that plants treated with this microalga showed greater accumulation of macro and micro elements. Herein, the different microalgae application methods successfully mitigated the adverse effects resulting from alkaline stress on the growth and productivity of chia plants 

### 4.5. Soil pH and Microbial Traits 

In this study, all microalgae applications caused an increase in the bacteria and cyanobacteria count and significantly reduced the pH levels in the experimental soil (Figure 3). This may be due to a rise in the number of microorganisms in the rhizosphere [100] and their impacts on the quantity of organic materials in microbial biomass [101], as well as microbial byproducts that contain extracellular polysaccharides and cell wall fragments [102]. Additionally, the application of microalgae improved soil biological activity since microalgae are a continuously replenishing source of carbon [103]. Kapil et al. [104] stated that cyanobacteria application is a significant strategy in bioremediation of alkaline soil as it declines the soil pH from 9.2 to 7.5. Among all microalgae applications, *C. vulgaris* showed the lowest cyanobacteria count, but with a significant increase relative to untreated soil. That may be due to their vital role in decreasing the soil pH (Figure 2), as generally, cyanobacteria prefer neutral pH for optimum growth and establishment [105].

In this study, *A. platensis* was superior to microalgae as an application spray or drenching method*. Arthrospira platensis* is a species of photosynthetic cyanobacteria with a high nutritional content [106], and there has been a growing interest in its biological activity and bioactive components [54]. Furthermore, *A. platensis* showed effects similar to those of IAA and gibberellin, which stimulate root elongation and establishment as well as accelerate vegetative growth [53,107]. Additionally, it is favorable for a variety of applications in the life sciences because it is Food and Drug Administration (FDA) certified as Generally Recognized as Safe (GRAS) [108], and can support special biological functions [109].

## 5. Conclusions

Microalgae application by the methods of foliar spray and soil drenching is a potential strategy for enhancing the growth and production of chia plants under alkaline stress conditions. As a great source of nutrients and growth hormones, the microalgae improved alkalinity resistance in chia by modulating soil, nutrient homeostasis, and photosynthetic effectiveness. Furthermore, the application of microalgae via different methods can greatly enhance seed and oil yields and optimize the plant’s composition in terms of total protein and carbohydrate contents, non-enzymatic compounds (phenolics and flavonoids), and DPPH, as well as alter fatty acid profiles. Therefore, applying various microalgae applications by foliar spray or soil drenching would be a potential and eco-friendly approach for enhancing agricultural productivity in alkaline environments. Our findings also suggest that the application of *Arthrospira platensis* using the soil drenching technique should be used in the future to enhance plant growth and productivity under alkaline soil conditions.

## Figures and Tables

**Table 1:** The physiochemical and biological properties of the experimental soil before cultivation.

Soil Characteristics	Value	
Particle Size Distribution (%)	2019/2020	2020/2021
Coarse sand	7.39	7.42
Fine sand	40.51	40.48
Silt	18.89	18.92
Clay	33.21	33.18
Texture class	Sandy clay Loam	
pH	7.94	7.92
ECe (dS m^–1^)	3.77	3.75
Soluble cations (mmolc L^−1^)		
Ca^++^	5.13	5.10
Mg^++^	4.12	4.10
Na^+^	28.22	28.08
K^+^	0.23	0.22
Soluble anions (mmolc L^−1^)		
CO_3_^–^	16.82	16.84
HCO_3_^–^	3.15	2.95
Cl^–^	16.28	16.30
SO_4_^–^	1.45	1.41
SAR	13.44	13.37
ESP%	15.65	15.58
Available Nutrients (%)		
N	0.17	0.18
P	0.20	0.22
K	0.29	0.30
Biological (CFU g^–1^ soil)		
Total bacterial count	7.15 × 10^7^	8.20 × 10^7^
Total cyanobacterial count	6.14 × 10^2^	6.89 × 10^2^

SAR, sodium adsorption ratio; ESP, exchangeable sodium percentage.

**Table 2:** Herb fresh and dry weights and total chlorophyll and carotenoid contents of chia plants cultivated under alkaline stress conditions in response to different application methods, microalgae strains, and their interaction.

Treatment		Herb Fresh Weight (g)	Herb Dry Weight (g)	Total Chlorophyll (SPAD)	Total Carotenoids (mg mm^−2^)
Season (S)					
S_I_		400.2 ± 0.07 b	101.2 ± 2.32 b	61.3 ± 2.91 b	0.39 ± b
S_II_		414.3 ± 0.05 a	106.3 ± 2.21 a	65.8 ± 2.90 a	0.47 ± a
Methods					
Spray		417.2 ± 3.11 a	105.0 ± 2.41 a	62.9 ± 2.88 a	0.43 ± 0.04 a
Drench		399.4 ± 3.14 b	100.3 ± 2.71 b	60.5 ± 2.67 b	0.36 ± 0.05 b
Microalgae					
Control		323.1 ± 0.90 d	80.5 ± 0.83 d	36.5 ± 0.22 e	0.23 ± 0.01 d
*Arthrospira* *platensis*		489.0 ± 3.40 a	123.3 ± 1.85 a	77.1 ± 0.67 a	0.68 ± 0.02 a
*Chlorella vulgaris*		438.6 ± 3.54 b	110.7 ± 1.63 b	72.1 ± 0.69 b	0.50 ± 0.03 b
*Nostoc muscorum*		394.2 ± 3.38 c	99.6 ± 0.84 c	63.9 ± 1.15 c	0.28 ± 0.01 c
*Anabaena azollae*		396.8 ± 3.08 c	99.4 ± 0.87 c	59.0 ± 0.46 d	0.28 ± 0.01 c
Methods × Microalgae					
Spray	Control	323.0 ± 1.17 g	81.1 ± 0.42 g	36.7 ± 0.03 i	0.23 ± 0.01 g
	*A. platensis*	505.2 ± 2.87 a	127.3 ± 0.72 a	78.2 ± 0.61 a	0.73 ± 0.01 a
	*C. vulgaris*	453.0 ± 1.27 c	114.3 ± 0.32 c	73.6 ± 0.37 c	0.58 ± 0.01 c
	*N. muscorum*	401.5 ± 0.89 e	101.4 ± 0.22 e	66.3 ± 0.60 e	0.30 ± 0.01 e
	*A. azollae*	403.5 ± 0.91 e	101.2 ± 0.55 e	59.8 ± 0.30 g	0.29 ± 0.01 e
Drench	Control	323.2 ± 1.64 g	80.0 ± 1.72 g	36.1 ± 0.39 i	0.22 ± 0.01 g
	*A. platensis*	472.8 ± 1.77 b	119.2 ± 0.47 b	75.9 ± 0.70 b	0.63 ± 0.01 b
	*C. vulgaris*	424.2 ± 2.10 d	107.0 ± 0.53 d	70.6 ± 0.28 d	0.43 ± 0.01 d
	*N. muscorum*	386.9 ± 1.68 f	97.7 ± 0.41 f	61.6 ± 0.88 f	0.26 ± 0.01 f
	*A. azollae*	390.2 ± 1.54 f	97.5 ± 0.39 f	58.3 ± 0.61 h	0.26 ± 0.01 f
*p*-value					
S		<0.001 ***	<0.001 ***	0.004 **	<0.001 ***
Methods		0.002 **	0.008 **	0.006 **	<0.001 ***
Microalgae		<0.001 ***	<0.001 ***	<0.001 ***	<0.001 ***
Methods × Microalgae		<0.001 ***	<0.001 ***	0.006 **	<0.001 ***

** and *** indicate differences at *p* ≤ 0.01 and ≤ 0.001 probability levels, and “ns” indicates non-significant difference. Mean values sharing the same lowercase letter for methods, microalgae, and their interaction in the same column do not differ significantly at *p* ≤ 0.05 by DMRT. Mean values are presented as mean ± standard error.

**Table 3:** Number of inflorescence plant ^−1^, seed yield plant^−1^, and 1000 seed weight of chia plants cultivated under alkaline stress conditions in response to different application methods, microalgae strains, and their interaction.

Treatment		No. Inflorescence Plant^−1^	Seed Yield Plant^−1^ (g)	1000 Seed Weight (g)
Season (S)				
S_I_		16.3 ± 1.31 b	14.56 ± 0.76 b	1.80 ± 0.16 b
S_II_		19.5 ± 1.22 a	16.78 ± 0.64 a	1.89 ± 0.12 a
Methods				
Spray		17.9 ± 1.50 a	14.40 ± 0.86 b	1.81 ± 0.11 b
Drench		15.2 ± 1.38 b	16.00 ± 0.1.11 a	1.86 ± 0.11 a
Microalgae				
Control		9.2 ± 0.83 d	9.59 ± 0.03 d	1.08 ± 0.03 e
*Arthrospira* *platensis*		24.4 ± 0.95 a	19.99 ± 0.69 a	2.22 ± 0.01 a
*Chlorella vulgaris*		20.3 ± 0.83 b	18.32 ± 0.45 b	2.13 ± 0.02 b
*Nostoc muscorum*		14.8 ± 0.71 c	14.19 ± 0.37 c	1.93 ± 0.02 c
*Anabaena azollae*		13.9 ± 0.75 c	13.91 ± 0.47 c	1.81 ± 0.03 d
Methods × Microalgae				
Spray	Control	10.2 ± 0.62 a	9.56 ± 0.03 f	1.08 ± 0.00 g
	*A. platensis*	26.2 ± 0.64 a	18.5 ± 0.05 b	2.19 ± 0.00 b
	*C. vulgaris*	21.5 ± 1.33 a	17.3 ± 0.09 c	2.09 ± 0.01 c
	*N. muscorum*	16.2 ± 0.52 a	13.51 ± 0.30 e	1.92 ± 0.03 d
	*A. azollae*	15.2 ± 0.58 a	13.07 ± 0.54 e	1.75 ± 0.00 f
Drench	Control	8.2 ± 0.52 a	9.61 ± 0.05 f	1.08 ± 0.01 g
	*A. platensis*	22.5 ± 0.86 a	21.49 ± 0.36 a	2.25 ± 0.01 a
	*C. vulgaris*	19.2 ± 0.60 a	19.30 ± 0.17 b	2.16 ± 0.02 b
	*N. muscorum*	13.4 ± 0.47 a	14.87 ± 0.39 d	1.94 ± 0.01 d
	*A. azollae*	12.6 ± 0.86 a	14.75 ± 0.32 d	1.87 ± 0.01 e
*p*-value				
S		0.013 *	<0.001 ***	<0.001 ***
Methods		0.017 *	<0.004 **	0.021 *
Microalgae		<0.001 ***	<0.001 ***	<0.001 ***
Methods × Microalgae		0.804 ^ns^	0.149 ^ns^	0.004 **

*, **, and *** indicate differences at *p* ≤ 0.05, ≤ 0.01, and ≤ 0.001 probability levels, and “ns” indicates non-significant difference. Mean values sharing the same lowercase letter for methods, microalgae, and their interaction in the same column do not differ significantly at *p* ≤ 0.05 by DMRT. Mean values are presented as mean ± standard error.

**Table 4:** The interactive effect of different application methods and various microalgae applications on the fatty acid profile of chia plants cultivated under alkaline stress.

N.	Fatty Acids (%)	Spray					Drench				
		Control	*A. platensis*	*C. vulgaris*	*N. muscorum*	*A. azollae*	Control	*A. platensis*	*C. vulgaris*	*N. muscorum*	*A. azollae*
1	Butyric (C4:0)	0.01	nd	nd	nd	nd	0.04	0.07	0.13	Nd	nd
2	Caproic (C6:0)	0.01	nd	nd	nd	0.07	0.24	0.11	0.06	Nd	0.14
3	Caprylic (C8:0)	0.01	nd	0.09	nd	0.06	0.16	0.04	0.14	Nd	0.15
4	Capric (C10:0)	0.05	0.04	0.12	0.19	0.14	0.11	0.08	0.11	Nd	0.21
5	Undecanoic (C11:0)	nd	nd	nd	4.02	nd	nd	Nd	nd	Nd	nd
6	Lauric (C12:0)	0.02	0.04	nd	4.01	0.21	0.11	0.08	0.08	Nd	nd
7	Myristic (C14:0)	0.04	0.07	nd	0.71	0.08	0.06	0.04	0.08	0.05	nd
8	Pentadecanoic (C15:0)	nd	0.04	nd	0.28	0.09	nd	0.05	nd	Nd	nd
9	Palmitic (C16:0)	6.98	5.98	7.01	6.81	6.82	6.34	6.91	7.68	6.89	7.65
10	Palmitoleic (C16:1)	0.09	nd	nd	nd	0.11	0.17	0.18	0.16	Nd	nd
11	Margaric (C17:0)	nd	nd	nd	nd	nd	nd	Nd	0.06	Nd	nd
12	Stearic (C18:0 n-9)	nd	0.24	nd	nd	nd	nd	Nd	nd	Nd	nd
13	Oleic (C18:1 n-9)	11.01	6.78	9.38	7.01	15.59	12.06	5.69	7.6	8.01	7.88
14	Linoleic (C18:2 n-6)	17.26	20.15	18.19	18.63	17.01	16.01	20.97	19.92	20.03	19.27
15	α-linolenic (C18:3 n-3)	57.24	60.02	61.71	58.34	59.10	55.47	63.8	61.38	58.01	61.53
16	γ-Linolenic (C18:3 n-6)	nd	4.05	2.11	nd	0.62	0.94	1.46	1.98	Nd	0.57
17	Arachidic (C20:0)	7.23	0.83	0.66	nd	0.10	8.24	0.45	0.35	7.01	1.52
18	Gadoleic (C20:1)	0.05	0.82	0.42	nd	nd	0.05	Nd	0.14	Nd	nd
19	Docosanoic (C22:0)	nd	0.94	0.31	nd	nd	nd	0.07	0.13	Nd	0.68
20	Tetracosanoic (C24:0)	nd	nd	nd	nd	nd	nd	Nd	nd	Nd	0.4
	Area total	100%									
	SFA	7.04	7.31	7.32	11.81	7.20	6.51	7.15	8.03	6.94	8.73
	MUFA	18.38	8.43	10.46	7.01	15.80	20.52	6.32	8.25	15.02	9.40
	PUFA	74.50	84.22	82.01	76.97	76.73	72.42	86.23	83.28	78.04	81.37
	n6/n3 ratio	0.30	0.40	0.33	0.32	0.30	0.31	0.35	0.36	0.35	0.32

Data are shown as percentages of the total methyl esters. SFA, saturated fatty acid; MUFA, monounsaturated fatty acid; PUFA, polyunsaturated fatty acid; nd, not determined.

**Table 5:** Total protein, total carbohydrate, total phenolic, total flavonoid, and DPPH levels in chia seeds cultivated under alkaline stress conditions in response to different application methods, microalgae strains, and their interaction.

Treatment		Total Protein	Total Carbohydrates	Total Phenolic	Total Flavonoids	DPPH
		(%)		(mg 100 g^−1^)	(mg 100 g^−1^)	(μg mL^−1^)
Season (S)						
S_I_		24.1 ± 0.73 b	36.1 ± 0.61 b	34.5 ± 2.06 b	15.7 ± 1.03 b	65.2 ± 0.92 b
S_II_		25.4 ± 0.98 a	37.5 ± 0.63 a	36.5 ± 2.10 a	17.6 ± 1.01 a	66.9 ± 0.81 a
Methods						
Spray		24.2 ± 0.89 b	36.3 ± 0.58 b	34.7 ± 2.04 b	15.8 ± 1.15 b	65.3 ± 0.86 b
Drench		25.2 ± 1.03 a	37.3 ± 0.75 a	36.3 ± 2.20 a	17.5 ± 1.26 a	66.8 ± 0.99 a
Microalgae						
Control		18.3 ± 0.06 d	33.9 ± 0.02 d	21.2 ± 0.05 e	9.64 ± 0.09 e	60.7 ± 0.19 d
*Arthrospira* *platensis*		25.7 ± 0.24 b	41.1 ± 0.46 a	44.5 ± 0.37 a	23.1 ± 0.39 a	70.9 ± 0.35 a
*Chlorella vulgaris*		23.6 ± 0.23 c	37.7 ± 0.23 b	40.6 ± 0.44 b	19.4 ± 0.46 b	68.3 ± 0.44 b
*Nostoc muscorum*		28.0 ± 0.34 a	35.7 ± 0.22 c	35.2 ± 0.45 d	15.9 ± 0.48 c	65.2 ± 0.54 c
*Anabaena azollae*		28.0 ± 0.36 a	35.7 ± 0.22 c	36.0 ± 0.60 c	15.3 ± 0.48 d	65.1 ± 0.39 c
Methods × Microalgae						
Spray	Control	18.3 ± 0.09 g	33.8 ± 0.04 g	21.1 ± 0.09 i	9.46 ± 0.12 i	60.7 ± 0.29 f
	*A. platensis*	25.1 ± 0.01 d	40.1 ± 0.00 b	43.7 ± 0.03 b	22.3 ± 0.07 b	70.2 ± 0.03 b
	*C. vulgaris*	23.1 ± 0.01 f	37.1 ± 0.01 d	39.7 ± 0.06 d	18.4 ± 0.07 d	67.2 ± 0.03 c
	*N. muscorum*	27.2 ± 0.03 b	35.2 ± 0.04 f	34.2 ± 0.07 h	14.2 ± 0.06 h	64.0 ± 0.46 e
	*A. azollae*	27.2 ± 0.01 b	35.2 ± 0.03 f	34.6 ± 0.05 g	14.8 ± 0.07 g	64.4 ± 0.44 e
Drench	Control	18.3 ± 0.10 g	33.9 ± 0.03 g	21.2 ± 0.01 i	9.81 ± 0.04 i	60.8 ± 0.32 f
	*A. platensis*	26.2 ± 0.02 c	42.2 ± 0.03 a	45.3 ± 0.15 a	23.9 ± 0.33 a	71.7 ± 0.21 a
	*C. vulgaris*	24.1 ± 0.01 e	38.2 ± 0.03 c	41.6 ± 0.17 c	20.4 ± 0.17 c	69.4 ± 0.16 b
	*N. muscorum*	28.7 ± 0.10 a	36.2 ± 0.01 e	36.2 ± 0.04 f	16.4 ± 0.10 f	66.3 ± 0.06 d
	*A. azollae*	28.8 ± 0.09 a	36.2 ± 0.02 e	37.3 ± 0.07 e	16.9 ± 0.03 e	65.7 ± 0.31 d
*p*-value						
S		<0.001 ***	<0.001 ***	<0.001 ***	<0.001 ***	<0.001 ***
Methods		0.004 **	<0.001 ***	<0.001 ***	0.002 **	<0.001 ***
Microalgae		<0.001 ***	<0.001 ***	<0.001 ***	<0.001 ***	<0.001 ***
Methods × Microalgae		<0.001 ***	<0.001 ***	<0.001 ***	<0.001 ***	0.011 *

*, **, and *** indicate differences at *p* ≤ 0.05, ≤ 0.01, and ≤ 0.001 probability levels, and “ns” indicates non-significant difference. Mean values sharing the same lowercase letter for methods, microalgae, and their interaction in the same column do not differ significantly at *p* ≤ 0.05 by DMRT. Mean values are presented as mean ± standard error.

**Table 6:** Mineral content (P, K, Ca^2+^, Fe^2+^, Mg^2+^, Zn^2+^, and Na^2+^) of chia seeds cultivated under alkaline stress conditions in response to different application methods, microalgae strains, and their interaction.

Treatment		P	K^+^	Ca^2+^	Fe^2+^	Mg^2+^	Zn^2+^	Na^+^
		(mg g^−1^ Dry Seed)						
Season (S)								
S_I_		3.38 ± 0.35 a	22.9 ± 1.11 b	11.2 ± 0.64 b	7.51 ± 0.35 b	6.72 ± 0.37 b	3.91 ± 0.21 b	2.00 ± 0.14 b
S_II_		3.41 ± 0.32 a	25.8 ± 1.14 a	13.1 ± 0.54 a	8.39 ± 0.42 a	7.95 ± 0.42 a	4.51 ± 0.18 a	2.13 ± 0.13 a
Methods								
Spray		3.37 ± 0.39 a	23.7 ± 1.17 b	11.4 ± 0.54 b	7.70 ± 0.36 b	6.81 ± 0.36 b	3.95 ± 0.17 b	2.12 ± 0.15 a
Drench		3.43 ± 0.28 a	25.3 ± 1.23 a	12.7 ± 0.71 a	8.30 ± 0.45 a	7.65 ± 0.48 a	4.20 ± 0.19 a	2.01 ± 0.15 b
Microalgae								
Control		2.15 ± 0.01 c	17.5 ± 0.15 e	9.13 ± 0.02 d	6.07 ± 0.02 d	5.53 ± 0.10 d	3.15 ± 0.01 d	3.08 ± 0.02 a
*Arthrospira platensis*		4.62 ± 0.23 a	30.7 ± 0.28 a	15.2 ± 1.05 a	10.4 ± 0.15 a	10.1 ± 0.38 a	5.07 ± 0.03 a	1.36 ± 0.05 e
*Chlorella vulgaris*		3.52 ± 0.19 ab	27.6 ± 0.47 b	13.8 ± 0.31 b	8.88 ± 0.34 b	7.64 ± 0.23 b	4.50 ± 0.17 b	1.87 ± 0.04 d
*Nostoc muscorum*		2.92 ± 0.04 bc	23.5 ± 0.53 c	11.1 ± 0.26 c	7.34 ± 0.08 c	6.46 ± 0.01 c	3.83 ± 0.04 c	1.94 ± 0.03 c
*Anabaena azollae*		3.80 ± 0.90 ab	23.2 ± 0.39 d	11.0 ± 0.25 c	7.30 ± 0.09 c	6.44 ± 0.13 c	3.83 ± 0.05 c	2.09 ± 0.02 b
Methods × Microalgae								
Spray	Control	2.17 ± 0.01 a	17.2 ± 0.04i	9.09 ± 0.02 a	6.06 ± 0.04 g	5.34 ± 0.06 h	3.14 ± 0.01 g	3.03 ± 0.02 a
	*A. platensis*	4.10 ± 0.01 a	30.1 ± 0.01 b	13.6 ± 1.67 a	10.1 ± 0.04 b	9.23 ± 0.06 b	5.01 ± 0.01 b	1.28 ± 0.06 a
	*C. vulgaris*	3.10 ± 0.01 a	26.6 ± 0.16 d	13.1 ± 0.02 a	8.11 ± 0.00 d	7.14 ± 0.04 d	4.11 ± 0.01 d	1.82 ± 0.06 a
	*N. muscorum*	2.84 ± 0.01 a	22.4 ± 0.12 g	10.5 ± 0.05 a	7.13 ± 0.01 f	6.18 ± 0.10 f	3.75 ± 0.03 f	1.90 ± 0.05 a
	*A. azollae*	4.66 ± 1.82 a	22.4 ± 0.26 g	10.5 ± 0.03 a	7.14 ± 0.01 f	6.15 ± 0.04 f	3.75 ± 0.06 f	2.04 ± 0.03 a
Drench	Control	2.13 ± 0.01 a	17.9 ± 0.03 h	9.16 ± 0.04 a	6.08 ± 0.03 g	5.73 ± 0.10 g	3.15 ± 0.02 g	3.12 ± 0.01 a
	*A. platensis*	5.14 ± 0.02 a	31.3 ± 0.01 a	16.9 ± 0.06 a	10.8 ± 0.03 a	10.9 ± 0.01 a	5.12 ± 0.02 a	1.43 ± 0.05 a
	*C. vulgaris*	3.95 ± 0.03 a	28.6 ± 0.21 c	14.4 ± 0.23 a	9.64 ± 0.06 c	8.15 ± 0.04 c	4.88 ± 0.06 c	1.91 ± 0.05 a
	*N. muscorum*	2.99 ± 0.01 a	24.7 ± 0.20 e	11.7 ± 0.07 a	7.47 ± 0.07 e	6.74 ± 0.04 e	3.91 ± 0.01 e	1.99 ± 0.01 a
	*A. azollae*	2.95 ± 0.01 a	23.9 ± 0.36 f	11.6 ± 0.04 a	7.54 ± 0.03 e	6.73 ± 0.01 e	3.92 ± 0.00 e	2.13 ± 0.01 a
*p*-value								
S		0.057 ^ns^	<0.001 ***	0.013 *	<0.001 ***	<0.001 **	<0.001 **	0.004 **
Methods		0.887 ^ns^	0.011 *	0.046 *	0.002 **	0.003 **	0.001 **	0.034 *
Microalgae		0.007 **	<0.0001 ***	<0.001 ***	<0.001 ***	<0.001 **	<0.001 **	<0.001 **
Methods × Microalgae		0.184 ^ns^	<0.001 ***	0.092 ^ns^	<0.001 ***	<0.001 **	<0.001 **	0.853 ^ns^

*, **, and *** indicate differences at *p* ≤ 0.05, ≤ 0.01, and ≤ 0.001 probability levels, and “ns” indicates non-significant difference. Mean values sharing the same lowercase letter for methods, microalgae, and their interaction in the same column do not differ significantly at *p* ≤ 0.05 by DMRT. Mean values are presented as mean ± standard error.

## Data Availability

Not applicable.
