# Correction: Youssef et al. Foliar Spray or Soil Drench: Microalgae Application Impacts on Soil Microbiology, Morpho-Physiological and Biochemical Responses, Oil and Fatty Acid Profiles of Chia Plants under Alkaline Stress. *Biology* **2022**, *11*, 1844

**DOI:** 10.3390/biology12020224

**Published:** 2023-01-31

**Authors:** Samah M. Youssef, Rasha S. El-Serafy, Kholoud Z. Ghanem, Abeer Elhakem, Azza A. Abdel Aal

**Affiliations:** 1 Horticulture Department, Faculty of Agriculture, Fayoum University, Fayoum 63514, Egypt; 2 Horticulture Department, Faculty of Agriculture, Tanta University, Tanta 31527, Egypt; 3 Department of Biological Science, College of Science and Humanities, Shaqra University, Shaqra, Riyadh 11961, Saudi Arabia; 4 Department of Biology, College of Sciences and Humanities, Prince Sattam Bin Abdulaziz University, Al-Kharj 11942, Saudi Arabia; 5 Soil Microbiology Department, Soils, Water and Environment Research Institute, Agricultural Research Center, Giza 12619, Egypt

## Text Correction

There were three errors in the original publication [1]. ** By mistake, we copied them from another study that we are currently testing **.

1. continuous illumination (16/8 h light/dark, 2000 lux)

2. 10,700× *g* RCF.

3. and dried overnight in a dry oven at 100 °C

A correction has been made to Section *2.3. Microorganisms Source and Culture*:

The cultures were incubated in a growth chamber under continuous illumination (24 h light, 2000 lux) and a temperature of 25 °C ± 2 °C for all strains except the mesophilic alga *A. platensis*, which was grown at 35 °C ± 2 °C. After 30 days of incubation, the algal suspension was used for the field experiments. To determine algal biomass, 100 mL of the algal culture was taken after 30 days of incubation, centrifuged at 10,000 rpm for 10 min, and the supernatant was discarded. The pellet was centrifuged for 10 min at 10,000 rpm after passing through three phases of resuspension in distilled water to wash it. The cleaned pellet was weighed, air dried; then, the dry weight was recorded and calculated as g/L. For the field experiments, 1 g/L of dried algal biomass was used.

The authors state that the scientific conclusions are unaffected. This correction was approved by the Academic Editor. The original publication has also been updated.
